# Controversial Flow Cytometry Monitoring of a Relapse Case of Pediatric T Cell Acute Lymphoblastic Leukemia: A Case Report

**DOI:** 10.3389/fmed.2022.858809

**Published:** 2022-03-22

**Authors:** Delia Codruţa Popa, Andreea Şerbănică, Radu Obrisca, Ionut Şerbănică, Letiţia Radu, Cristina Jercan, Andra Marcu, Ana Bica, Minodora Asan, Mădălina Petran, Mihaela Dragomir, Cerasela Jardan, Valeria Ţică, Anca Gheorghe, Irina Stoian, Daniel Coriu, Anca Coliţă, Andrei Coliţă

**Affiliations:** ^1^Department of Medicine, Carol Davila University of Medicine and Pharmacy, Bucharest, Romania; ^2^Department of Hematology, Fundeni Clinical Institute, Bucharest, Romania; ^3^Department of Toxicology, Grigore Alexandrescu Emergency Clinical Hospital for Children, Bucharest, Romania; ^4^Department of Hematology, Coltea Hospital, Bucharest, Romania

**Keywords:** leukemia, T cell, children, relapse, allotransplant, stem cell

## Abstract

Acute lymphoblastic leukemia (ALL) is the most frequent childhood cancer, with 80–85% represented by B cell ALL and only 15% by T cell ALL. T Cell ALL (T-ALL) carries a more reserved prognosis compared to B Cell ALL (B-ALL) with regard to response to treatment, risk of relapse, and overall survival. Progress made in current monitoring protocols such as *via* flow cytometry immunophenotyping (FCM) and by PCR-based amplification of antigen-receptor genes led to improved management of patients with ALL and superior rates of survival. Nevertheless, challenges remain in some clinical cases. This manuscript describes a unique case of T-ALL and raises awareness of such clinical challenges. The article presents an overview of the flow cytometry immunophenotyping at diagnosis and during treatment of a pediatric patient with T-ALL from Fundeni Clinical Institute. In this case, in spite of various therapeutic measures such as first-line chemotherapy for high risk group, salvage chemotherapy (FLAG), conditioning regimen (FLU-BU-TT-ATG), and stem cell transplant, a chemoresistance clone continued to be present.

## Introduction

Acute lymphoblastic leukemia (ALL) is the most frequent childhood malignancy; 80–85% of cases originate from B cell progenitors, and only 15% from T-cell precursors (1). Over the past few decades, with current advances in treatment, survival and cure rates of childhood ALL have improved significantly. However, 10–15% of patients relapse and have overall inferior outcome. Frequent assessment of treatment response is crucial to evaluate the sensitivity of leukemic cells to therapy and adjust management accordingly. Previously, treatment response was monitored by morphological examination of bone marrow aspirates, an approach with limited sensitivity and specificity that can fail to detect residual leukemic cells. Thus, relying on morphology to detect residual disease leads to under-treatment and increased risk of relapse. Nowadays, minimal residual disease (MRD) using FCM and PCR base amplification of antigen receptor genes are more reliable and sensitive than morphological evaluation, and have a crucial role in determining risk stratification (2) resulting in improved cure rates in childhood ALL. T cell ALL represents one of the most challenging forms of pediatric ALL. Not only the monitoring of treatment response is more difficult than in B-ALL but these patients have rather limited therapeutic options. Thus, there are less opportunities to personalize therapy in patients with T-ALL and thus, there are higher relapse rates and worse morbidity and mortality compared to B ALL.

A 7-year-old male patient, without preexisting medical conditions, and no significant family history, presented with fatigue, appetite loss, multiple bilateral cervical adenopathies, and abdominal distension for 1 week prior to admission. Physical exam revealed facial edema with painful palpebral edema and chemosis. The patient showed skin pallor and petechiae limited to the anterior thorax and thighs. There was lymphadenopathy, hepatosplenomegaly, and a mediastinal mass. Initial complete blood count (CBC): WBC: 314 × 10^9^/L, Hb = 6.7 g/dl, PLT = 24 × 10^9^/L, with 89% blast cells on peripheral blood smear. Morphological examination of the bone marrow identified 90% L1 (FAB) lymphoblasts. Molecular analysis showed no mutation in E2A-PBX1-t(1; 19) (q23; p13), MLL-AF4-t(4;11) (q21; q23), BCR-ABLp190-t(9; 22) (q34; q11), BCR-ABL p210-t(9; 22) (q34; q11), TEL-AML1–t(12; 21) (p13; q22), and SIL-TAL 1-del (1) (p32; p32).

**Figure 1:**
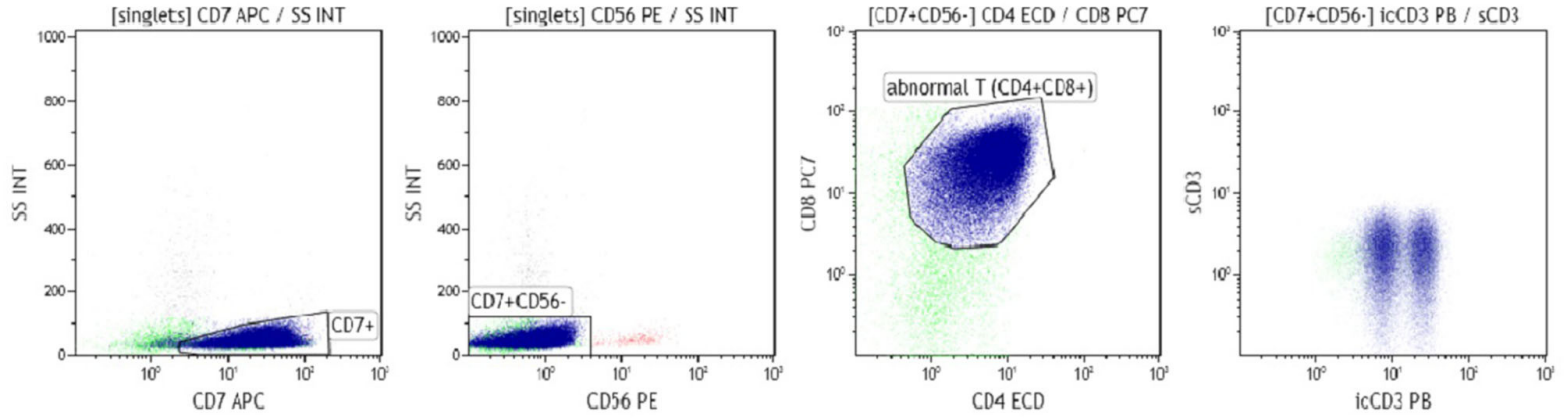
Immunophenotyping performed on bone marrow aspirate at diagnosis (Fundeni Clinical Institute archive).

Flow cytometry immunophenotyping of the bone marrow (BM) aspirate identified lymphoblasts cells based on CD45-/+weak that were positive for CD7, intracytoplasmatic CD3, CD5 (weak), CD4, and CD8, and were negative for surface CD3, CD16, CD56, CD19, CD20, CD10, CD13, CD14, CD33, anti-MPO, and HLA-DR (Figure 1).

Corroborating anamnestic, clinical, and laboratory data, a diagnosis of ALL-L1 (FAB) with cortical T cell was established, and the patient was initiated on treatment according to the ALL-BFM 2009 protocol.

**Figure 2:**
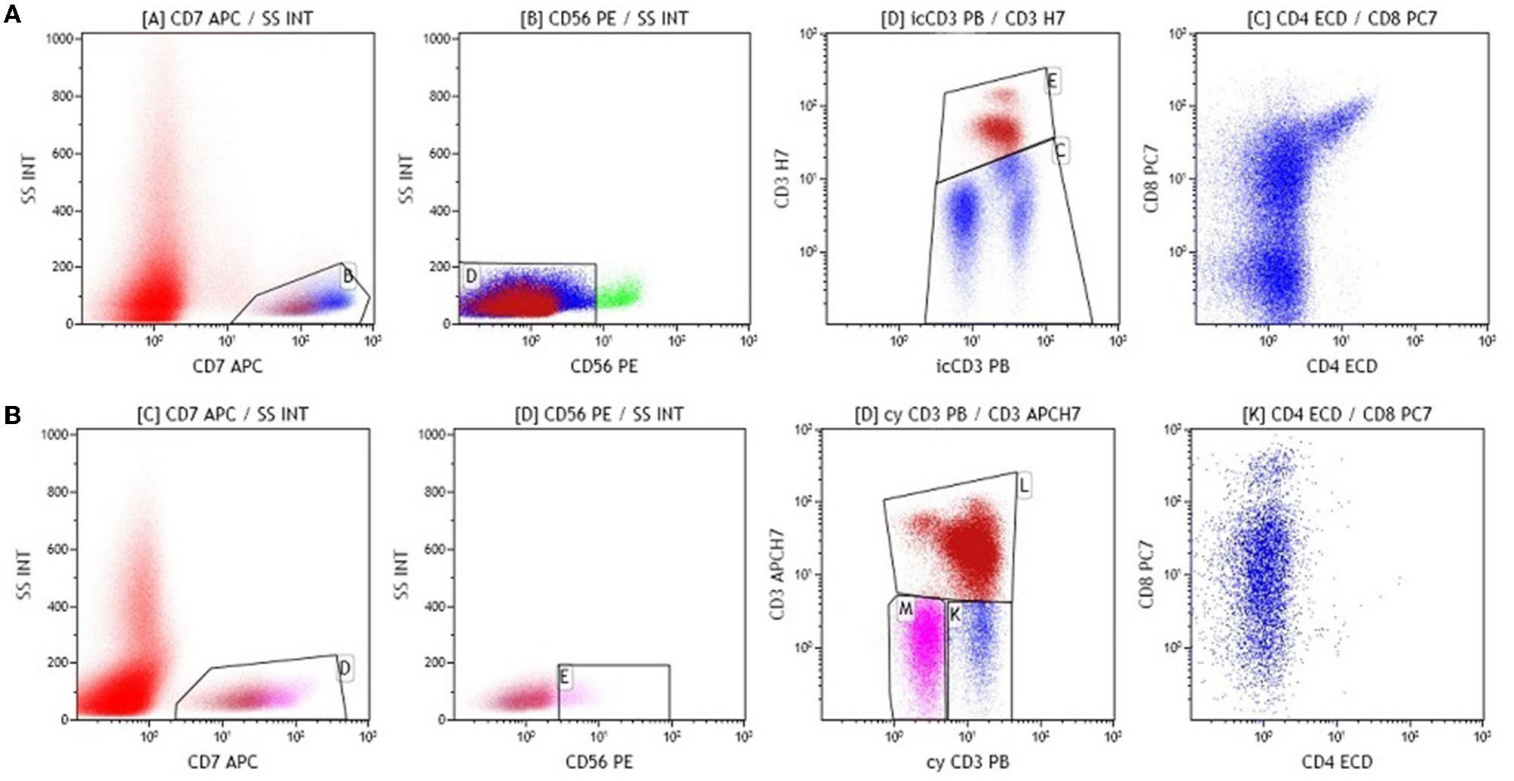
Immunophenotyping performed on bone marrow **(A,B)**.

Day 8 evaluation revealed good response to prednisone. FCM MRD_day15_ evaluation showed 8.8% atypical T lymphoblasts. The lymphoblasts had slight phenotypic changes compared to those at diagnosis. A fraction of blasts displayed a more mature T cell phenotype: surface CD3 + weak, CD4-, CD8 + weak/- (Figure 2A).

Subsequently, FCM MRD evaluation on day 33 performed at the end of the first part of induction therapy showed 0.9% atypical T lymphoblasts. The patient the continued treatment according to ALL-IC BFM 2009 protocol high-risk group (HRG) with close monitoring of MRD *via* FCM (Figure 2B).

**Figure 3:**
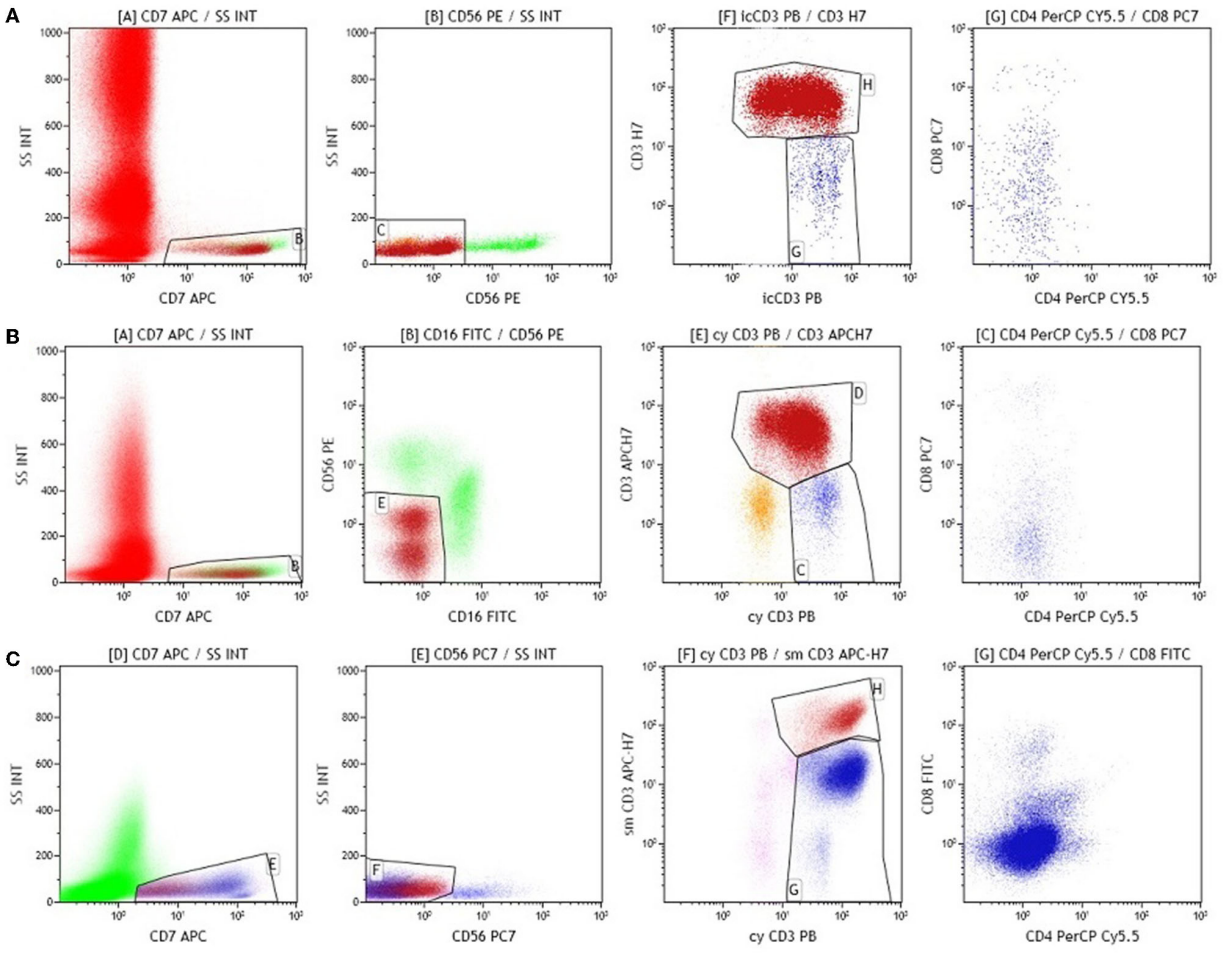
Immunophenotyping performed on bone marrow **(A–C)**.

On day 78, FCM MRD revealed 0.03% T lymphoblasts, similar to those found on day 33 (Figure 3A).

FCM monitoring of atypical T lymphoblasts was performed on the peripheral blood and the bone marrow aspirate. We observed a slight increase in atypical T lymphoblasts during monthly evaluation. The next FCM MRD_HRG1_ showed 0.14% atypical T lymphoblasts with major overall phenotypic alterations (CD7+CD56-CD16-icCD3+sCD3-CD4-CD8-/+weak) compared to those at diagnosis (CD7+CD56-CD16-icCD3+sCD3-CD4+CD8+). We hypothesized that the patient experienced hematological relapse with a clone distinct from that present at diagnosis and, thus, an unfavorable response to therapy (Figure 3B). Since multiple studies have shown that presence of complete remission (CR) before stem cell transplant is associated with improved overall survival rates (3), the patient was initiated on FLAG salvage chemotherapy to achieve MRD-negative remission. After the FLAG therapy, hematologic remission was achieved, but FCM MRD remained positive (0.53%). At this time, the patient proceeded with stem cell transplant. He underwent conditioning with Fludarabine-Busulfan-Thiotepa-ATGAM (FLU-BU-TT-ATG) followed by hematopoietic stem cell transplantation (HSCT), 10/10 HLA match unrelated donors. Patient received 5 × 10^6^ CD 34/kg peripheral blood stem cells and primary graft vs. host disease prophylaxis with cyclosporine (dose 5 mg/kg/day) beginning on day-1 until day 20 and methotrexate (MTX) 15 mg/sq on day +1 and 10 mg/sq on day +3, +6, +11. Patient engrafted on day 14 for PLT (38000/mm^3^) and on day 17 for neutrophils (ANC = 2.1 × 10^∧^9/l). The engraftment was documented by neutrophil count over 500/mm3 for three consecutive days. The whole blood chimerism was 87% donor DNA, and bone marrow flow cytometry showed 13.97% presence of atypical T lymphoblasts. Immunosuppressive treatment was discontinued on day +20, but donor chimerism continued to drop because of increasing percentage of T lymphoblasts consistent with progressive disease. In addition to disease progression, the patient also showed signs of CNS relapse (Figure 3C). He received 5 days nelarabine salvage therapy (650 mg/sq) and donor lymphocyte infusion (DLI) 1 × 10^6^ CD_3_/kg on day +30, with an unfavorable outcome. He died of progressive disease on day+35.

## Discussion

T-cell acute lymphoblastic leukemia (T-ALL) is a form of leukemia with a reserved prognosis compared to B-ALL regarding response to treatment, relapse risk, and survival rate. Although this patient had cortical T-ALL, which is considered the most favorable prognostic form among other forms of T-cell ALL (2), it showed primary resistance to initial therapy and unfavorable outcome. The European Group for the Immunophenotyping of T lymphoblasts recognizes the challenges faced by creating a consensus in this disease. Among the variables taken into account, number of cells acquired from BM, flow cytometry instrument, software with which cells are identified, presence/absence of various expression markers, changes in antigen expression during and after therapy, and experience of laboratory physicians are most likely to impact disease monitoring of T-ALL.

In an effort to standardize the nomenclature of this disease, the European Group for the Immunologic Classification of Leukemia proposed a classification of T-ALL into pro-T (cCD3^+^, sCD3^−^, CD1a^−^, CD2^+^, CD5^−^, CD7^+^, CD34^−^, CD4^−^, and CD8^−^), pre-T/immature (cCD3^+^, sCD3^−^, CD1a^−^, CD2^+^, CD5^+^, CD7^+^, CD34^−^, CD4^−^, and CD8), cortical T (cCD3^+^, sCD3^+/−^, CD1a^+^, CD2^+^, CD5^+^, CD7^+^, CD34^−^, CD8^+^, and CD4^+^), mature-T (cCD3^+^, sCD3^+^, CD1a^−^ CD2^+^, CD5^+^, CD7^+^, CD34^−^, CD8^+^, or CD4+), and ETP-ALL (early-T precursor), lack of CD1a and CD8 expression, weak CD5 expression, and expression of at least one myeloid and/or stem cell marker.

Studies suggested that cortical T-ALL has the best prognosis, and that early forms of T-ALL (ETP) has worse outcomes. Current advances in modern chemotherapy and MRD-based risk stratification may have blunted the prognostic value of maturation stage in T-ALL (4).

Of note, this case also showed that under treatment pressure secondary clones, as represented by atypical T lymphoblasts emerged and they now dictate overall prognosis in this disease (Table 1).

**Table 1:** Clonal evolution at multiple time points during the disease course.

**FCM time points**	**Dynamic MRD •Measurement • Phenotype**	**Therapeutic measure**
FCM at diagnosis	Approximately 90% T lymphoblast **CD7+, icCD3+, CD4+, CD8+**	ALL-IC 2009
MRD_day15_	3% more mature atypical T lymphoblasts **sCD3+weak, CD4-, CD8+weak/-**	ALL-IC 2009 Phase IA
MRD_day33_	0.9% atypical T lymphoblast **CD7++CD56-icCD3-/+heterogenousCD4-CD8-/+heterogenous**	ALL-IC 2009 Phase IB
MRD_day78_	0.03% atypical T lymphoblast **CD7++CD56-icCD3-/+heterogenousCD4-CD8-/+heterogenous**	ALL-IC 2009 High Risk
MRD_HRG_	0.14% atypical T blasts **CD7+CD56-CD16-icCD3+sCD3-CD4-CD8-/+weak**	salvage therapy FLAG
MRD_afterBMT_	0.53% atypical T blasts infiltration **CD7+CD56-CD16-icCD3+sCD3-CD4-CD8-/+weak** 13.9% atypical T blasts infiltration **CD7+CD56-CD16-icCD3+sCD3-CD4-CD8-/+weak**	Before conditioning therapy FLU-BU-TT-ATG Day+20 after AlloHSCT Salvage 5 days NELARABINE monotherapy
Peripheral blood+BM	Massive atypical T blasts infiltration	Patient died of progressive disease Day+35

T-ALL has higher relapse risk compared to B-ALL. In our case, the patient presented new clone, after the period of induction (5). Usually, medullary relapse is associated with unfavorable prognosis and requires aggressive chemotherapy followed by bone marrow transplantation (6).

In this case, in spite of aggressive chemotherapy with FLAG, MRD-negative remission was not possible, and the subsequent BMT was not sufficient to overcome this poor prognostic factor. The patient did not resume normal hematopoiesis post-transplant, and the early engraftment was accompanied by reconstitution with atypical T lymphoblasts, the hallmark of the leukemic population identified by MRD on day 15.

The process of relapse in pediatric T-cell acute lymphoblastic leukemia is a multistep event that results in increased genomic complexity that alters mechanisms that control cell cycle, proliferation, and differentiation of lymphocytes (7).

In one study, Kunz et al. showed two different pathways of relapse in T-ALL: about half of relapse samples were characterized by the presence of additional mutations on top of mutations present in a diagnostic clone, while in the other half of relapse samples the clone driving relapse is not derived from a major diagnostic clone but rather from a minor ancestral one with totally new mutations (8). Although patients with T-cell ALL are at higher risk of relapse and have worse mortality and morbidity rates, studies showed a survival rate of only 30–40% even after relapse, with the condition of obtaining second remission (9). A number of agents targeting T cell antigens *via* either chimeric antigen receptor (CAR) T or NK cells, naked antibodies such as daratumumab (10) or bispecific antibodies are under clinical development for patients with T-ALL. Thus, enrolling these patients in clinical trials is a top priority. In this regard, CAR T cells targeting various T cell lineage antigens, such as CD3, CD4, CD5, and CD7, have shown potent cytotoxicity against the T ALL cell line and primary tumors *in vitro*. Nevertheless, challenges such as selection of an appropriate antigen, the isolation of sufficient number of non-leukemic T cells, lymphoblast contamination in autologous T cell products, long-term aphasia) (11) remain to be overcome in their path toward clinical development.

## Conclusion

The T cell phenotype is associated with worse treatment outcomes and multiple difficulties in FCM MRD monitoring. In our case, extreme therapeutic measures included high-risk chemotherapy (3 blocks) achievement of without achieving CR, together with salvage chemotherapy (FLAG), conditioning treatment (FLU-BU-TT-ATG), and stem cell allotransplantation procedure that demonstrated the presence of an increased new chemoresistance clone.

## Data Availability Statement

The original contributions presented in the study are included in the article/supplementary material, further inquiries can be directed to the corresponding authors.

## Ethics Statement

The studies involving human participants were reviewed and approved by Fundeni Clinical Institute Ethics Committee. Written informed consent was obtained from the participants' legal guardian/next of kin to participate in this study and for the publication of any potentially identifiable images or data included in this article.

## Author Contributions

DP and AŞ: conceptualization, methodology, investigation, and writing original draft preparation. RO, ISe, LR, CJe, AM, AB, MA, MP, MD, CJa, VŢ, AG, and ISt: methodology, investigations, collected data, data interpretation, and preparation of the manuscript. AncC, DP, AŞ, and DC: validation, writing review, and editing. AndC: supervised the work. All authors contributed to the article and approved the submitted version.

## Conflict of Interest

The authors declare that the research was conducted in the absence of any commercial or financial relationships that could be construed as a potential conflict of interest.

## Publisher's Note

All claims expressed in this article are solely those of the authors and do not necessarily represent those of their affiliated organizations, or those of the publisher, the editors and the reviewers. Any product that may be evaluated in this article, or claim that may be made by its manufacturer, is not guaranteed or endorsed by the publisher.
